# The Impact of *ARMS2* (rs10490924), *VEGFA* (rs3024997), *TNFRSF1B* (rs1061622), *TNFRSF1A* (rs4149576), and *IL1B1* (rs1143623) Polymorphisms and Serum Levels on Age-Related Macular Degeneration Development and Therapeutic Responses

**DOI:** 10.3390/ijms25179750

**Published:** 2024-09-09

**Authors:** Dzastina Cebatoriene, Alvita Vilkeviciute, Greta Gedvilaite-Vaicechauskiene, Monika Duseikaite, Akvile Bruzaite, Loresa Kriauciuniene, Dalia Zaliuniene, Rasa Liutkeviciene

**Affiliations:** 1 Medical Academy, Lithuanian University of Health Sciences, A. Mickeviciaus St. 9, LT-44307 Kaunas, Lithuania; dzastina.cebatoriene@lsmu.lt; 2 Neuroscience Institute, Medical Academy, Lithuanian University of Health Sciences, Eiveniu St. 2, LT-50161 Kaunas, Lithuania; greta.gedvilaite@lsmu.lt (G.G.-V.); monika.duseikaite@lsmu.lt (M.D.); akvile.bruzaite@lsmu.lt (A.B.); loresa.kriauciuniene@lsmu.lt (L.K.); rasa.liutkeviciene@lsmu.lt (R.L.); 3 Department of Ophthalmology, Medical Academy, Lithuanian University of Health Sciences, Eiveniu St. 2, LT-50161 Kaunas, Lithuania; dalia.zaliuniene@lsmu.lt

**Keywords:** age-related macular degeneration, gene polymorphisms, *ARMS2* (rs10490924), *IL1B1* (rs1143623), *TNFRSF1B* (rs1061622), *TNFRSF1A* (rs4149576), *VEGFA* (rs3024997), ARMS2, IL1B1, TNFRSF1B, TNFRSF1A, VEGFA, ELISA, anti-VEGF therapy

## Abstract

Age-related macular degeneration (AMD) is a major global health problem as it is the leading cause of irreversible loss of central vision in the aging population. Anti-vascular endothelial growth factor (anti-VEGF) therapies are effective but do not respond optimally in all patients. This study investigates the genetic factors associated with susceptibility to AMD and response to treatment, focusing on key polymorphisms in the *ARMS2* (rs10490924), *IL1B1* (rs1143623), *TNFRSF1B* (rs1061622), *TNFRSF1A* (rs4149576), *VEGFA* (rs3024997), ARMS2, IL1B1, TNFRSF1B, TNFRSF1A, and VEGFA serum levels in AMD development and treatment efficacy. This study examined the associations of specific genetic polymorphisms and serum protein levels with exudative and early AMD and the response to anti-VEGF treatment. The AA genotype of VEGFA (rs3024997) was significantly associated with a 20-fold reduction in the odds of exudative AMD compared to the GG + GA genotypes. Conversely, the TT genotype of *ARMS2* (rs10490924) was linked to a 4.2-fold increase in the odds of exudative AMD compared to GG + GT genotypes. In females, each T allele of *ARMS2* increased the odds by 2.3-fold, while in males, the TT genotype was associated with a 5-fold increase. Lower serum IL1B levels were observed in the exudative AMD group compared to the controls. Early AMD patients had higher serum TNFRSF1B levels than controls, particularly those with the GG genotype of *TNFRSF1B* rs1061622. Exudative AMD patients with the CC genotype of *TNFRSF1A* rs4149576 had lower serum TNFRSF1A levels compared to the controls. Visual acuity (VA) analysis showed that non-responders had better baseline VA than responders but experienced decreased VA after treatment, whereas responders showed improvement. Central retinal thickness (CRT) reduced significantly in responders after treatment and was lower in responders compared to non-responders after treatment. The T allele of *TNFRSF1B* rs1061622 was associated with a better response to anti-VEGF treatment under both dominant and additive genetic models. These findings highlight significant genetic and biochemical markers associated with AMD and treatment response. This study found that the *VEGFA* rs3024997 AA genotype reduces the odds of exudative AMD, while the *ARMS2* rs10490924 TT genotype increases it. Lower serum IL1B levels and variations in TNFRSF1B and TNFRSF1A levels were linked to AMD. The *TNFRSF1B* rs1061622 T allele was associated with better anti-VEGF treatment response. These markers could potentially guide risk assessment and personalized treatment for AMD.

## 1. Introduction

Age-related macular degeneration (AMD) is one of the most common causes of blindness in developed countries. Therefore, AMD significantly impacts quality of life, leading to difficulties in performing daily tasks, loss of independence, and increased risk of depression. The primary risk factor for AMD is age, with the severity of vision impairment ranging from mild to severe. Individuals over the age of 75 face a 25% risk of developing early AMD and an 8% risk of progressing to late AMD, with the number of cases expected to increase due to the aging population [1]. AMD leads to pathological changes in the deeper retinal layers of the macula and the surrounding blood vessels, resulting in a loss of central vision. The accumulation of deposits on the retina, called drusen, is a characteristic clinical finding in AMD and may be the first sign of the “dry” form of the disease. Dry AMD is the most common morphological type. It can progress to “wet” or neovascular AMD [1]. The neovascular, or “wet”, form of AMD is less common but accounts for 90% of cases of acute blindness caused by AMD [2]. Globally, the prevalence of advanced AMD is estimated to be 1.6% [3]. Neovascular AMD (nAMD) is characterized by the formation of neovascular choroidal membranes, exudation, and fibrosis, leading to acute vision loss [4]. Intravitreal injection of an anti-vascular endothelial growth factor is the standard therapy to maintain or improve visual acuity in most patients with nAMD. While numerous studies have shown positive outcomes with anti-VEGF therapies, there are limitations to their use. Previous studies indicate that 20% of patients continue to experience vision loss, and approximately 50% fail to achieve visual acuity 20/40 [5,6]. Despite the efficacy of anti-VEGF agents in many cases, some patients exhibit an incomplete response to treatment, characterized by persistent intraretinal or subretinal fluid and ongoing vision loss [7]. As AMD is a multifactorial condition, identifying its risk factors allows individuals to make informed lifestyle choices that may lower their risk of developing the disease. Ongoing genetic studies in AMD should prioritize determining the mechanism, pathways, and networks underlying the disease, not just the risk factors including aging, smoking, and high blood pressure, so that appropriate pathways for treatment and efficacy can be identified. Many risk factors play a role in the development of AMD but the most important role is played by genetic variants, which were identified over the years in different studies, suggesting complex oligogenic patterns of inheritance for AMD [8,9,10,11,12,13,14,15,16,17].

In the GWAS experiment [18], the identified genes were found to function in known AMD pathways and highlight the importance of additional pathways. These include complement activation, collagen synthesis, lipid metabolism and cholesterol transport, receptor-mediated endocytosis, endodermal cell differentiation, and extracellular matrix organization [18]. Researchers conducting a large genome-wide association study have identified 52 coding variants in 34 loci that occur more frequently in patients with AMD [18]. The genetic/poligenetic risk score (GRS/PRS) is important to explore in different cohorts as its calculation depends on the presence of risk variants that may be differently distributed across populations. Genetic and epidemiological research has established the undeniable role of genetic variation in the etiology of AMD, with the heritable component estimated to be between 45% and 70% [19]. This is especially true when integrating genetic information with other interacting environmental and demographic factors to better predict disease risk [20,21,22,23]. Geographic variations might explain the discrepancies and a specific genetic variant was found to be more common in Western Europe compared to other global regions, justifying its superior prevalence and effect in the Portuguese population [24]. The complex etiology of AMD depends not only on the genetic background and is greatly impacted and modified by environmental factors [20,21].

While the primary loci associated with advanced AMD are concentrated around the complement factor H gene on chromosome 1q31, recent large-scale genome-wide association studies have increasingly confirmed associations with other genes that may be risk factors [25]. Polymorphisms of the age-related maculopathy 2 gene (*ARMS2*, also known as *LOC387715*), located at the 10q26 locus, have been strongly associated with the inverse effect of hormone replacement therapy on AMD. Researchers have demonstrated that the mRNA and corresponding peptide encoded by ARMS2 are expressed in the retina, indicating that the *ARMS2* transcript is responsible for the association with AMD [26]. In addition, the ARMS2 protein has been described as a component of the extracellular matrix. Its mRNA has a unique splice form in the retina that is not found in other tissues [27]. The polymorphic site A69S (rs10490924) within the *ARMS2* locus has been investigated in several studies, mainly concerning AMD, and the results showed a strong correlation between poor visual acuity in advanced AMD and response to anti-VEGF intervention [28].

The human vascular endothelial growth factor A (*VEGFA*) gene is located on chromosome 6, with multiple common SNPs in the promoter and the 5′ and 3′ untranslated regions. It is organized into eight exons and seven introns [29]. VEGFA is a member of the VEGF-related polypeptide family and plays a key role in increasing vascular permeability, angiogenesis, endothelial cell growth, and migration [30]. Vascular leakage and inflammation caused by the excessive release of VEGFA have been found to play a crucial role in choroidal neovascularization and the development of neovascular AMD. VEGFA and its signaling pathway have been targeted at the pathogenic processes in which they are involved in the most effective therapeutic development. Polymorphisms in the *VEGFA* gene regulate VEGF expression and thus its angiogenic properties [31]. It is, therefore, reasonable to suggest that different expression levels of VEGF may result in different responses to anti-VEGF drugs. Finally, a study examining seven different *VEGFA* polymorphisms concluded that none of these significantly predicts the success of anti-VEGF treatment with Bevacizumab in patients with nAMD [32].

Through interaction with its receptors TNFR1 and TNFR2, the ligand of tumor necrosis factor α (TNFα) activates the inflammatory response, cell proliferation, and differentiation [33,34]. Several studies have shown that TNFR1 and TNFR2 receptor isoforms respond differently to TNF-α stimulation and anti-TNF-α therapy [35]. However, despite the accumulated data on the specifics of the functioning and regulation of TNFR1 and TNFR2 signaling pathways, these remain incompletely understood.

The IL-1 family of cytokines is key in triggering acute inflammatory responses [36]. IL-1β interacts with the IL-1 receptor I (IL-1RI), which consists of the IL-1R and IL-1R accessory protein subunits. The IL-1 receptor antagonist (IL-1Ra) competes with IL-1β for its binding site [36]. IL-1β is a potent inflammatory mediator with chemotactic and angiogenic properties [37,38]. It is a neurotoxic mediator in ischemic brain injury but can attenuate glutamate neurotoxicity in the retina and protect against light-induced or hereditary photoreceptor degeneration [39,40,41]. In AMD, IL-1β is secreted by retinal pigment epithelial (RPE) cells and CD68+ cells in choroidal neovascular membranes and is, therefore, a potential pro-angiogenic and neuroprotective or neurotoxic mediator in AMD [42].

Our research aims to evaluate the roles of *ARMS2* (rs10490924), *VEGFA* (rs3024997), *TNFRSF1B* (rs1061622), *TNFRSF1A* (rs4149576), and *IL1B1* (rs1143623), as well as the serum levels of these genetic markers, in the development of AMD and the efficacy of its treatment.

## 2. Results

Our current study involved 253 patients diagnosed with early AMD, 245 patients with exudative AMD, and 337 healthy controls. The control group was formed of 337 subjects that matched gender classification in the early and exudative AMD group structure; however, subjects of the control group were younger than the exudative AMD patients (*p* < 0.001), and further analysis was performed and adjusted by age (Table 1). 

### 2.1. Hardy–Weinberg Equilibrium Analysis

A quality assessment based on Hardy–Weinberg equilibrium (HWE) analysis showed that the distribution of genotypes of *VEGFA* rs3024997, *IL1B* rs1143623, *TNFRSF1B* rs1061622, *TNFRSF1A* rs4149576, and *ARMS2* rs10490924 did not deviate from HWE in the control group (*p* < 0.05).

### 2.2. VEGFA rs3024997, IL1B rs1143623, TNFRSF1B rs1061622, TNFRSF1A rs4149576, and ARMS2 rs10490924 Associations with Early and Exudative AMD

The frequencies of genotypes and alleles for the following SNPs were analyzed within the study groups: *VEGFA* rs3024997, *IL1B* rs1143623, *TNFRSF1B* rs1061622, *TNFRSF1A* rs4149576, and *ARMS2* rs10490924.

For *VEGFA* rs3024997 (GG, GA, and AA), we observed a statistically significant difference between the exudative AMD and the control groups, with frequencies of 63.3%, 36.3%, and 0.4% in exudative AMD, respectively, compared to 55.5%, 38.3%, and 6.2% in the control group (*p* < 0.001). Furthermore, the A allele was less frequent in the exudative AMD group, accounting for 18.6% compared to 25.4% in the control group (*p* = 0.006). Similarly, for *ARMS2* rs10490924 (GG, GT, and TT), we found a statistically significant difference between the exudative AMD and control groups, with frequencies of 31.8%, 43.3%, and 24,9%, compared to 54.3%, 38.3%, and 7.4% (*p* < 0.001). The T allele was more frequent in the exudative AMD group, accounting for 46.5% compared to 26.6% in the control group (*p* < 0.001) (Table 2). 

Binary logistic regression analysis was conducted to assess the impact of selected SNPs on both early and exudative AMD. No statistically significant results were observed for the early AMD group after applying Bonferroni correction (Supplementary Table S1). However, in the exudative AMD group, *VEGFA* (rs3024997) exhibited a significant association. In the most robust model, the AA genotype was found to reduce the odds of exudative AMD by approximately 20-fold compared to the GG + GA genotypes (OR = 0.049, 95% CI: 0.006–0.381; *p* = 0.004). Similarly, *ARMS2* (rs10490924) showed a significant association in the most robust model, where the TT genotype was associated with 4.2-fold increased odds of exudative AMD compared to the GG + GT genotypes (OR = 4.236, 95% CI: 2.508–7.155; *p* = 0.004) (Table 3).

#### Analysis of *VEGFA* rs3024997, *IL1B* rs1143623, *TNFRSF1B* rs1061622, *TNFRSF1A* rs4149576, and *ARMS2* rs10490924 in Early and Exudative AMD in Female and Male Subgroups

We observed a statistically significant difference in the distribution of *ARMS2* rs10490924 genotypes (GG, GT, and TT) between females with exudative AMD and the control groups, with frequencies of 30.3%, 46.5%, and 23.2% compared to 54.1%, 38.3%, and 7.7%, respectively (*p* < 0.001). Additionally, the T allele was more prevalent in the exudative AMD females’ group, accounting for 46.5% compared to 26.8% in the control group (*p* < 0.001) (Table 4).

Binary logistic regression analysis was performed to evaluate the impact of selected SNPs on females with early and exudative AMD. No statistically significant results were observed for early AMD females after applying Bonferroni correction (Supplementary Table S2). Meanwhile, in the exudative AMD group, *ARMS2* (rs10490924) showed a significant association in the most robust genetic model, where each T allele was associated with 2.3-fold increased odds of exudative AMD in females (OR = 2.265 (1.617–3.172); *p* = 0.004) (Table 5).

Also, we observed a statistically significant difference in the distribution of *ARMS2* rs10490924 genotypes (GG, GT, and TT) between males with exudative AMD and the control groups, with frequencies of 34.4%, 37.8%, and 27.8% compared to 54.1%, 38.3%, and 7.7%, respectively (*p* < 0.001). Additionally, the T allele was more prevalent in the exudative AMD male group, accounting for 46.7% compared to 26.1% in the control group (*p* < 0.001) (Table 6).

Binary logistic regression analysis was performed to evaluate the impact of selected SNPs on males with early and exudative AMD. No statistically significant results were observed after applying Bonferroni correction for males with early AMD (Supplementary Table S3). Meanwhile, in males with exudative AMD, *ARMS2* (rs10490924) showed a significant association in the most robust model, where the TT genotype was associated with about 5-fold increased odds of exudative AMD in males compared to the GG + GT genotypes (OR = 5.049 (2.147–11.877); *p* < 0.001) (Table 7).

### 2.3. Serum IL1B, TNFRSF1B, TNFRSF1A, and ARMS2 Associations with Early and Exudative AMD

**Figure 1:**
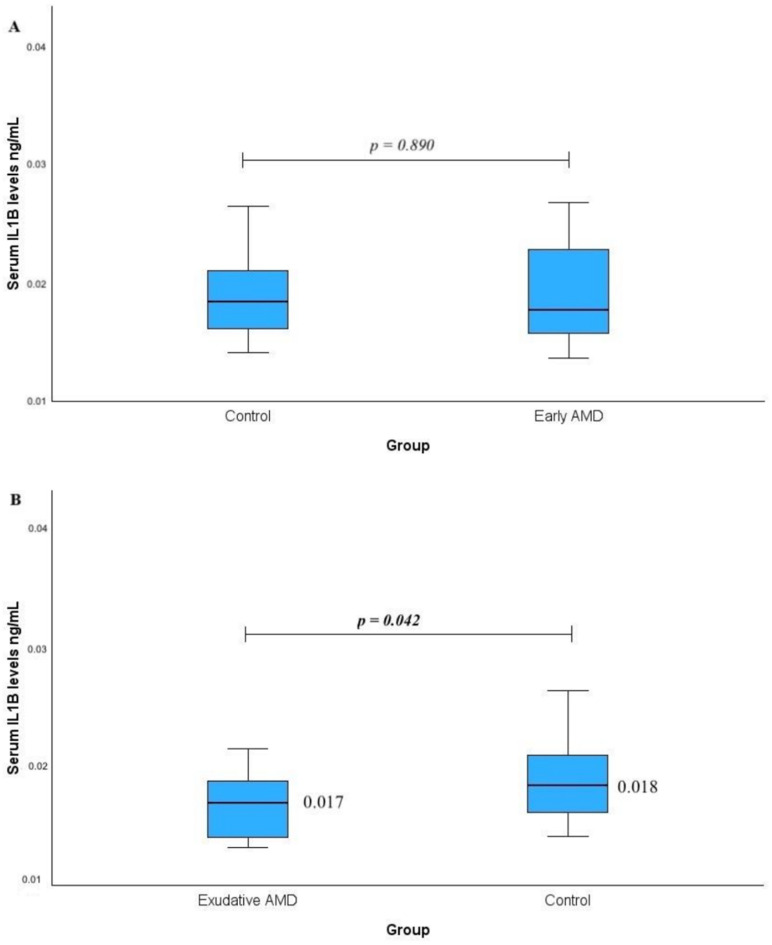
Serum IL1B levels were measured in patients with early AMD vs. control group (**A**) and exudative AMD vs. control groups (**B**). *p*-values marked with bold indicate statistically significant *p*-values, significant when *p* < 0.05; Mann–Whitney U test was used.

Serum IL1B levels were measured in patients with early AMD vs. the control group (A) and in patients with exudative AMD vs. the control group (B). No statistically significant difference was found when comparing early AMD with the control group (median (IQR): 0.018 (0.008) vs. 0.018 (0.005), *p* = 0.890). However, statistically significantly lower serum IL1B levels were observed in the exudative AMD group compared to the controls (median (IQR): 0.017 (0.005) vs. 0.018 (0.005), respectively; *p* = 0.042, with a medium effect size, r_rb_ = 0.323). The results are shown in Figure 1.

**Figure 2:**
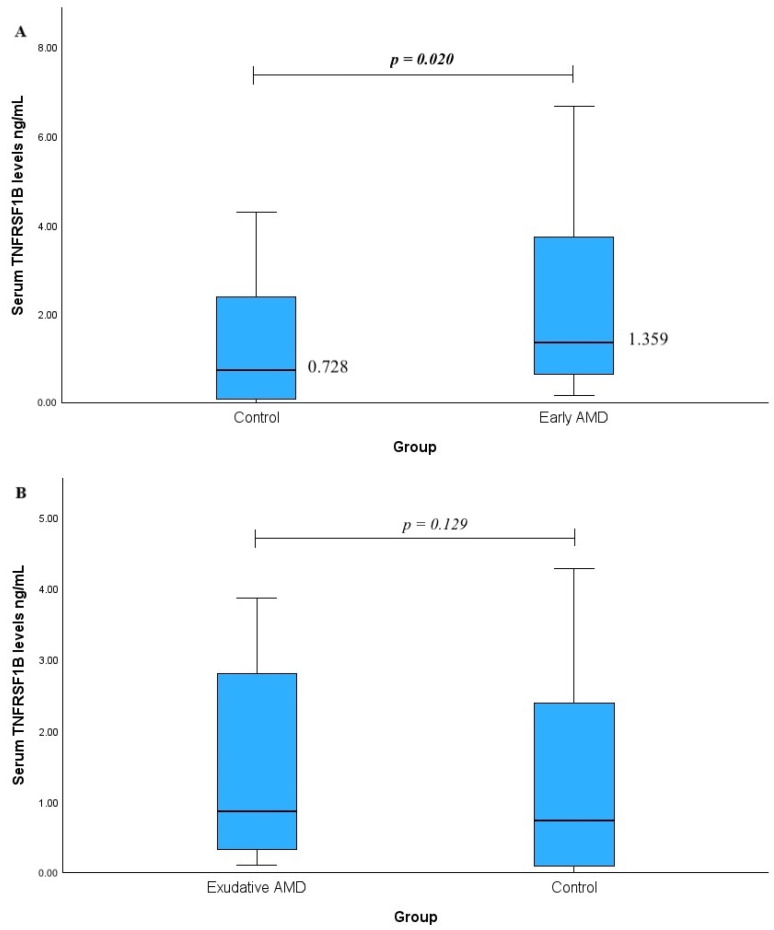
Serum TNFRSF1B levels were measured in patients with early AMD vs. control group (**A**) and exudative AMD vs. control group (**B**). *p*-values marked with bold indicate statistically significant *p*-values, significant when *p* < 0.05; Mann–Whitney U test was used.

Serum TNFRSF1B levels were measured in patients with early AMD versus the control group (A) and in patients with exudative AMD versus the control group (B). A statistically significant difference was observed in the early AMD group (median (IQR): 1.359 (4.012) vs. 0.728 (2.331); *p* = 0.020, with a medium effect size, r_rb_ = 371). However, no statistically significant difference was found when comparing exudative AMD with the control group (median (IQR): 0.858 (3.014) vs. 0.728 (2.331), *p* = 0.129). The results are shown in Figure 2.

Serum TNFRSF1A levels were measured in patients with early AMD vs. the control group (A) and in patients with exudative AMD vs. the control group (B). However, no statistically significant difference was found when comparing early AMD with the control group (median (IQR): 0.517 (0.662) vs. 0.436 (0.432), *p* = 0.627) or exudative AMD with the control group (median (IQR): 0.393 (0.633) vs. 0.436 (0.432), *p* = 0.883) (Supplementary Figure S1).

Serum ARMS2 levels were measured in patients with early AMD vs. the control group (A) and in patients with exudative AMD vs. the control group (B). However, no statistically significant difference was found when comparing early AMD with the control group (median (IQR): 0.220 (0.274) vs. 0.268 (0.224), *p* = 0.155) or exudative AMD with the control group (median (IQR): 0.152 (0.358) vs. 0.268 (0.224), *p* = 0.163) (Supplementary Figure S2).

### 2.4. Serum IL1B, TNFRS1B, TNFRS1A, and ARMS2 Levels and IL1B, TNFRS1B, TNFRS1A, and ARMS2 SNP Associations with AMD

**Figure 3:**
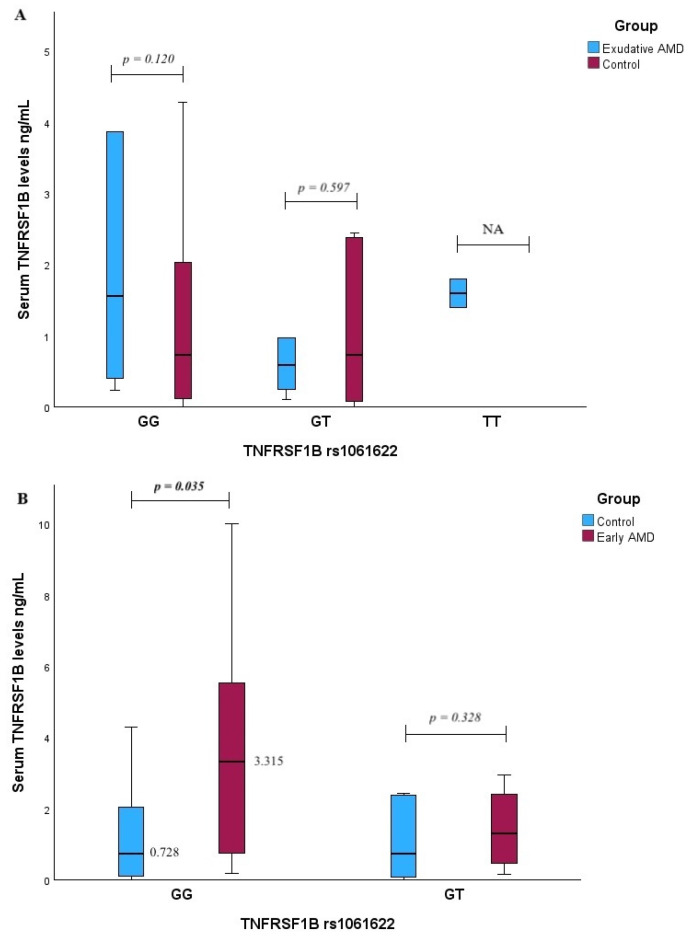
Serum TNFRSF1B levels were measured in patients with exudative AMD vs. control group and compared between *TNFRSF1B* rs1061622 genotypes (**A**) and between early AMD vs. control group (**B**). *p*-values marked with bold indicate statistically significant *p*-values, significant when *p* < 0.05; Mann–Whitney U test was used.

Serum IL1B, TNFRS1B, TNFRS1A, and ARMS2 levels were compared among different genotypes for selected single-nucleotide polymorphisms. No statistically significant IL1B levels and *IL1B* rs1143623 or ARMS2 and *ARMS2* rs10490924 genotype associations were revealed with early and exudative AMD occurrence (Supplementary Figures S3 and S4). However, early AMD patients with the GG genotype of *TNFRS1B* rs1061622 exhibited higher serum TNFRS1B levels compared to the control group (median (IQR): 3.315 (6.853) vs. 0.728 (2.460), *p* = 0.035, with a medium effect size, r_rb_ = 0.412) (Figure 3B).

**Figure 4:**
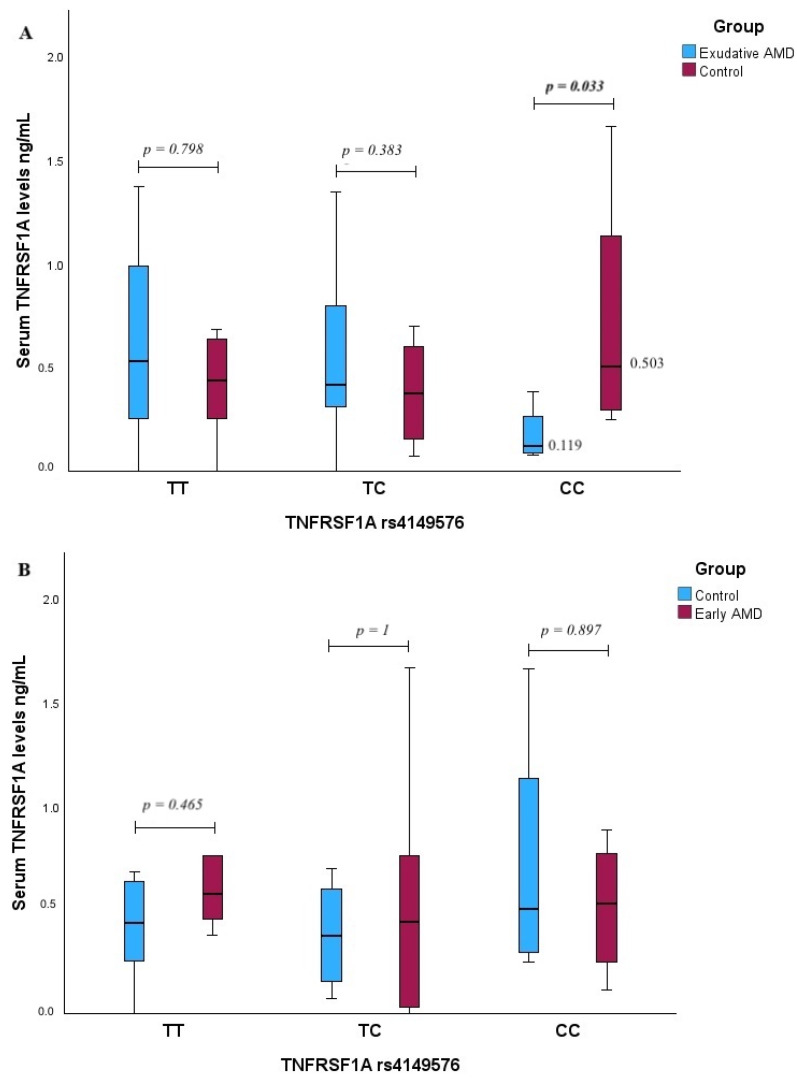
Serum TNFRSF1A levels were measured in patients with exudative AMD vs. control group and compared between *TNFRSF1A* rs4149576 genotypes (**A**) and between early AMD vs. control group (**B**). *p*-values marked with bold indicate statistically significant *p*-values, significant when *p* < 0.05; Mann–Whitney U test was used.

The analysis of TNFRS1A serum levels among different genotypes of *TNFRS1A* rs4149576 revealed that exudative AMD patients with the CC genotype exhibited lower serum TNFRS1A levels compared to the control group (median (IQR): 0.119 (0.241) vs. 0.503 (0.982), *p* = 0.033, with a large effect size, r_rb_ = 0.833) (Figure 4A). 

### 2.5. Response to Exudative AMD Treatment with Anti-VEGF Injections

The treatment response was evaluated for 115 patients with exudative AMD. The demographic and response to treatment parameters of the study group are summarized in Table 8. There was no difference in age or gender distribution between non-responders and responders.

We compared the median visual acuity (VA) between non-responders and responders using the Mann–Whitney U test, and the analysis showed that non-responders at the baseline had better VA than responders (0.465 (0.45) vs. 0.28 (0.26), *p* = 0.018, with a medium effect size, r_rb_ = −0.336). On the other hand, while comparing the VA and CRT data before and after treatment using Wilcoxon signed-rank test, the VA was decreased after the treatment (0.465 (0.45) vs. 0.35 (0.35), *p* = 0.028, with a large effect size, r_rb_ = −0.567) in non-responders and improved in responders (0.28 (0.26) vs. 375 (35), *p* < 0.001, with a medium effect size, r_rb_ = −0.442) after the treatment.

The median CRT was significantly lower in responders than in non-responders after treatment (274 (95) vs. 329 (103), *p* = 0.032, with a medium effect size, r_rb_ = 0.440). Also, CRT decreased after treatment statistically significantly only in responders (320.5 (113) vs. 274 (95), *p* < 0.001, with a strong effect size, r_rb_ = −0.072), but not in non-responders) (Table 8).

### 2.6. Single-Nucleotide Polymorphism Associations with Exudative AMD Treatment Response

Binomial logistic regression analysis was performed to analyze the association between all SNPs and anti-VEGF treatment response, and only one SNP was found to be linked to treatment response with anti-VEGF. The *TNFRSF1B* rs1061622 T allele was found to be associated with a better response to anti-VEGF treatment under the dominant (OR  =  4.302; 95% CI: 1.181–15.674; *p* = 0.027) and additive (OR  =  3.999; 95% CI: 1.176–13.602; *p* = 0.026) genetic models (Table 9).

### 2.7. Serum IL1B, TNFRSF1B, TNFRSF1A, and ARMS2 Associations with the Treatment Response to Anti-VEGF Treatment

A comparison of serum IL1B, TNFRSF1B, TNFRSF1A, and ARMS2 was conducted among non-responders and responders but no statistically significant differences were observed comparing these groups (*p* > 0.05). VEGFA serum concentrations were measured and described in our previous publication [43] but did not show statistical differences between study groups.

## 3. Discussion

Age-related macular degeneration causes irreversible vision loss, and targeted anti-vascular endothelial growth factor therapy is now the most common and effective treatment. This paper aims to discuss whether genetic polymorphisms of *ARMS2* (rs10490924), *VEGFA* (rs3024997), *TNFRSF1B* (rs1061622), *TNFRSF1A* (rs4149576), and *IL1B1* (rs1143623) and their serum biomarkers could confer susceptibility to early and exudative AMD with a response to anti-VEGF treatment. 

Most patients with active neovascular AMD are offered treatment with intravitreal anti-VEGF agents, such as Bevacizumab (Avastin, Genentech, South San Francisco, CA, USA), Ranibizumab (Lucentis, Genentech, South San Francisco, CA, USA), Aflibercept (Eylea, Regeneron, Tarrytown, NY, USA), Brolucizumab (Beovu, Novartis, Basel, Switzerland), and Faricimab (Vabysmo, Genentech, South San Francisco, CA, USA). Considerable diversity in treatment response among patients is evident, with some individuals responding more favorably to specific anti-VEGF agents than others. This suggests the presence of a pharmacogenetic effect [44,45]. Research findings suggest a higher prevalence of AMD among first-degree relatives of affected individuals, with an odds ratio of 2.4 [46]. Clinical genetic testing is an important part of emerging personalized medicine [47]. Improved risk stratification through clinical genetic testing can enhance patient outcomes by optimizing resource allocation and facilitating the selection of the most suitable treatment options [48,49]. Genetic variants can account for up to 70% of the clinical variability in AMD, indicating the potential for personalized medical approaches to address this disease effectively [18,48,50]. 

Suboptimal responses and limited effectiveness over time can significantly impact patients, leading to poor vision outcomes. Despite the positive outcomes seen in many patients, around 25–35% of individuals with nAMD experience suboptimal responses to current anti-VEGF treatments, encounter delayed treatment failure or necessitate intensive and frequent IVT therapy [51,52]. Additionally, the necessity for recurrent treatments for nAMD poses a considerable burden on healthcare systems, patients, and their caregivers. Moreover, while current anti-VEGF treatments are generally effective, they are also associated with certain adverse events that can significantly impact eyesight, albeit infrequently. For instance, endophthalmitis, a severe complication, occurs in approximately 1 in 3500 injections [53]. Another significant potential complication of anti-VEGF therapy is the risk of intraocular inflammation. In severe cases, this inflammation can lead to irreversible vision loss [54]. Furthermore, a temporary increase in intraocular pressure is frequently noticed shortly after IVT injection of all anti-VEGF agents. Additionally, a temporary increase in intraocular pressure is commonly observed shortly after intravitreal injection of all anti-VEGF agents [55].

Repeated use of anti-VEGF treatments can also lead to adverse effects. For instance, macular atrophy, an advanced phenotype of nAMD, potentially linked to long-term anti-VEGF use, may result in permanent vision loss [56]. Among the 35% who do not respond optimally to therapy, over 10% experience deterioration despite treatment, while an additional 25% show no signs of improvement [7].

Gene therapy for nAMD is challenging due to the complexity of the genes associated with the condition.

### 3.1. Genetic Variants and AMD

In our study, we found potential genetic links to AMD, specifically exudative AMD.

***VEGFA* rs3024997 and *ARMS2* rs10490924**: Our analysis demonstrated a significant difference in genotype and allele frequencies of *VEGFA* rs3024997 and *ARMS2* rs10490924 between exudative AMD patients and healthy controls. Specifically, the AA genotype of *VEGFA* rs3024997 was associated with a reduced risk of exudative AMD, while the TT genotype of *ARMS2* rs10490924 was linked to an increased risk. These findings corroborate previous studies indicating the role of these SNPs in AMD susceptibility. A Japanese study reported that the SNP rs699946 in the *VEGFA* gene was associated with a better visual response after 12 months of treatment with bevacizumab [57]. An alternative study concluded that the SNP rs3025000 correlated with enhanced visual outcomes following six months of anti-VEGF treatment [58].

Polymorphisms in the *VEGFA* gene regulate VEGF expression and, thus, its angiogenic properties [59]. Therefore, it is possible that differing levels of VEGF expression could lead to varied responses to anti-VEGF drugs. Studies have explored genetic variations within the *VEGFA* and *VEGFR2* genes in small cohorts to understand their influence on anti-VEGF treatment outcomes, showing mixed results. For example, one study noted a trend toward better visual outcomes after six months of ranibizumab treatment in patients with the risk genotype for the *VEGFA* SNP rs1413711 compared to those with the non-risk genotype [60]. A recent study investigating two *VEGFA* SNPs and their relationship with ranibizumab response found that rs699947 influences early functional outcomes [61]. However, other genetic variations related to blood vessel growth regulation may still be linked to treatment response [59]. Zhang and colleagues analyzed 21 studies to examine the link between the *ARMS2* gene and the response to anti-VEGF treatment in advanced AMD. They found that individuals with the G allele for *ARMS2* A69S had a more favorable outcome with anti-VEGF drugs, especially among East Asian patients. Further validation through large clinical trials is needed [62].

***TNFRSF1A* and *TNFRSF1B***: Our results also revealed significant associations between *TNFRSF1B* rs1061622 and exudative AMD, where the GG genotype was associated with higher serum levels of TNFRSF1B and increased odds of AMD. This aligns with the literature suggesting that *TNFRSF1B* can influence inflammatory processes and cell death, pivotal in AMD pathogenesis [63,64,65,66,67]. TNFRSF1A and TNFRSF1B are two proteins that belong to the TNF-receptor superfamily. They are mainly secreted by macrophages and can induce cell death of specific tumor cell lines. They are also potent pyrogens that can cause fever by direct action or stimulating interleukin-1 secretion. Moreover, under certain conditions, they can promote cell proliferation and differentiation [68]. The proteins encoded by these genes form a heterocomplex with TNF-receptor 1, which mediates the recruitment of two anti-apoptotic proteins. Although their function is not entirely understood, they are believed to be associated with anti-apoptotic signals. Knockout studies in mice have also suggested that these proteins protect neurons from apoptosis by stimulating antioxidative pathways. The role of TNFRSF1A in AMD remains unclear, but its association with other diseases and SNPs highlights its potential involvement in AMD [69,70,71].

**IL1B**: Our study found that IL1B’s association with AMD was less pronounced than in other studies. We observed significantly lower IL1B levels in the exudative AMD group compared to the controls, with no significant associations found with specific SNPs in *IL1B*. This contrasts with other studies linking *IL1B* with AMD through its inflammatory role [72,73]. IL-1β, a pro-inflammatory cytokine, plays a crucial role in immune responses, inflammation, and various disease processes. It has been implicated in retinal degenerative diseases and choroidal neovascularization (CNV). Studies have shown that inhibition of IL-1β can ameliorate these conditions [74]. Furthermore, IL-1β has also been associated with abnormal angiogenesis processes, indicating its role in promoting diverse cellular signaling pathways. The pro-inflammatory cytokine IL-1β has been shown to promote angiogenesis and can have a neurotoxic or neuroprotective effect. To determine whether IL-1β plays a role in CNV and retinal degeneration, the team conducted a study to analyze the expression of IL-1β in mice with laser-induced CNV and light-induced retinal degeneration in rats and mice [75]. IL-1β is induced in acute and chronic brain degenerative diseases and the retina. This association could result from the activation of inflammation, which further stimulates RPE cells to trigger photoreceptor degeneration and neovascularization. IL-1β was involved in the abnormal angiogenesis process. This could activate different abnormal angiogenesis processes through distinct cell signaling pathways. The variability in findings may be attributed to different abnormal angiogenesis processes through distinct cell signaling pathways, as well as differences in study populations or methodologies [39].

### 3.2. Serum Biomarkers and AMD

**IL1B, TNFRSF1A, and TNFRSF1B**: IL1B levels were significantly lower in exudative AMD patients than in the controls, though no significant differences were found between early AMD and controls. The lower levels of IL1B in exudative AMD patients might reflect a complex interplay of inflammatory processes rather than a straightforward biomarker of AMD severity. TNFRSF1B levels were significantly higher in early AMD patients than controls but not in exudative AMD patients. This suggests a potential differential role of TNFRSF1B in early versus advanced stages of AMD. This aligns with the proposed protective role of TNFRSF1B in neuronal apoptosis and its varying effects across disease stages [70,71]. In published databases, we did not uncover any studies examining the serum correlations of TNFRSF1A, TNFRSF1B, and IL1B1 with AMD. 

**ARMS2 and VEGFA**: Despite the known genetic associations with AMD risk, we did not find significant differences in serum ARMS2 levels between AMD patients and controls. This may indicate that ARMS2’s contribution to AMD is more strongly associated with genetic factors than circulating protein levels. Battu et al. [76] found that ARMS2 serum levels were significantly elevated in the AMD group compared to the control group. The highest levels of ARMS2 and VEGF proteins were recorded for the wet AMD sub-group. The study results endorsed a significant positive correlation between the following molecules: ARMS2 and VEGF (r = 0.925, *p* < 0.0001), COL8A1 and VEGF (r = 0.879, *p* < 0.0001), and RAD51B and VEGF (r = 0.691, *p* < 0.0001) [76]. VEGFA serum levels, as well as *VEGFA* SNPs, are widely studied in AMD patients. While we did not find any statistical differences in VEGF-A serum levels between the exudative AMD patients and controls, we confirmed the results from several other studies, which included total AMD patients or only exudative AMD patients, consisting of 27 to 71 samples per group in different populations [76,77,78,79,80,81,82,83]. 

### 3.3. Response to Anti-VEGF Treatment

Our study also examined the response to anti-VEGF treatment in exudative AMD patients. Responders had improved visual acuity and reduced CRT post-treatment compared to non-responders. This is consistent with the expected clinical outcomes of effective anti-VEGF therapy. Interestingly, non-responders initially had better baseline visual acuity, which suggests that pre-treatment visual acuity might influence the response to treatment. However, the lack of significant differences in serum biomarkers between responders and non-responders indicates that these markers may not be reliable predictors of treatment efficacy.

## 4. Materials and Methods

### 4.1. Ethics

This study adhered to the guidelines of the Declaration of Helsinki and received approval from the Kaunas Regional Biomedical Research Ethics Committee of the Lithuanian University of Health Sciences (approval number BE-2-/48). Informed consent was obtained from all participants.

### 4.2. Study Design and Structure

The study subjects were admitted to the Ophthalmology Department at the Hospital of the Lithuanian University of Health Sciences in Kaunas from 2014 to 2023 and underwent an ophthalmological evaluation. Information about their health and other diseases was collected during examinations by general practitioners and from medical records.

### 4.3. SNP Selection

The single-nucleotide polymorphisms (SNPs) investigated in our study were carefully selected based on their different and various associations with disease mechanisms. We thoroughly reviewed other research papers on these polymorphisms and their links to various diseases. After extensive analysis, we identified the most relevant SNPs to study in relation to age-related macular degeneration, its stages, and potential treatments. 

Many researchers have investigated AMD from the perspective of the whole genome. A potent technique for locating genetic variations that may be linked to AMD is whole exome sequencing (WES), which is performed on genomic areas of the genome that code for proteins. Typically, the goal of this research is to find both common and uncommon genetic variations that may affect the disease’s course or chance of acquiring AMD. Identifying novel risk loci, validating established genetic connections, and gaining insights into the molecular mechanisms underlying AMD are some of the major outcomes of whole exome studies in AMD, as *ARMS2*, *VEGFA*, *TNFRSF* [84,85,86], and *IL1B1* were selected based on association with the pathogenetic mechanism of the chronic inflammatory process [87].

### 4.4. Study Group Formation

All study participants underwent a comprehensive ophthalmological evaluation during which data on general health and comorbidities were systematically collected. Informed consent was obtained from each participant prior to their inclusion in the study. The participants were then stratified into two groups: those diagnosed with age-related macular degeneration (AMD) and the control subjects. AMD patients were assessed by an ophthalmologist following previously established guidelines [88]. Detailed inclusion and exclusion criteria for both AMD patients and control subjects have been meticulously outlined in our earlier publication [88].

### 4.5. Exudative AMD Response to Anti-VEGF Injection Treatment

The efficacy of anti-VEGF treatments (Ranibizumab, Aflibercept, Bevacizumab) was evaluated in patients with exudative age-related macular degeneration (AMD) characterized by exudative or hemorrhagic macular features. Patients who had not previously received intravitreal anti-VEGF injections were followed for three to six months post-treatment. Central macular thickness (CMT) and best corrected visual acuity (BCVA) were measured before treatment and at three and six months afterward. Patients were categorized as responders or non-responders based on clinical OCT and BCVA data. Detailed methods and criteria for response evaluation, including definitions of structural changes, visual acuity deterioration, and patient categorization, were described in a previous publication [88].

### 4.6. DNA Extraction from Peripheral Venous Blood and Genotyping

Deoxyribonucleic acid (DNA) extraction and genotyping of selected single-nucleotide polymorphisms (SNPs)—*VEGFA* rs3024997, *IL1B* rs1143623, *TNFRSF1B* rs1061622, *TNFRSF1A* rs4149576, and *ARMS2* rs10490924—were performed at the Laboratory of Ophthalmology, Neuroscience Institute, Lithuanian University of Health Sciences, using predesigned TaqManTM Genotyping assays (Thermo Fisher Scientific, Pleasanton, CA, USA) following the manufacturer’s recommendations.

### 4.7. Serum Protein Concentration Measurement

To prepare the serum, blood was drawn from peripheral veins and allowed to incubate at room temperature for 30 min before being centrifuged. After centrifugation, the serum was carefully separated from the cell pellet, transferred into 2 mL tubes, and stored at −80 °C until analysis. The levels of IL1B, TNFRSF1B, TNFRSF1A, and ARMS2 in the serum of both AMD patients and control subjects were measured according to the manufacturer’s instructions.

However, the VEGFA serum concentrations were measured and described in our previous publication [43].

### 4.8. Statistical Analysis

The statistical analysis was conducted using SPSS/W 29.0 software (Statistical Package for the Social Sciences for Windows, Inc., Chicago, IL, USA). The normality of continuous data (age, BCVA, and CMT) was assessed using the Shapiro–Wilk test. Continuous variables that did not follow the normal distribution model were expressed as median with interquartile range (IQR) and compared using the non-parametric Mann–Whitney U test. Normally distributed data were expressed as mean with standard deviation (SD) and compared using the Student T test. The nonparametric Wilcoxon signed-rank test was used to compare differences in the BCVA and CMT before and after treatment. Statistical significance was defined as *p* < 0.05. Rank-biserial correlation (r_rb)_ was used to measure the effect size of particular results.

Categorical data (sex and genotype distributions) were presented as absolute numbers with percentages in parentheses and compared between groups using the Pearson’s chi-squared test (χ^2^). 

The influence of gene polymorphisms on early and exudative AMD was evaluated using binomial logistic regression analysis. We ensured that the key assumptions underlying our binomial regression analysis were satisfied. Specifically, the analysis was conducted on a dichotomous dependent variable, the independence of observations was maintained by design with no repeated measures or clustering, and the sample size was sufficient to yield stable and reliable estimates. Therefore, we confirm that the assumptions for the applied binomial regression analysis were adequately met. The results were presented as odds ratio (OR) with a 95% confidence interval (CI), adjusted for age in the exudative AMD groups. Genetic models (codominant, dominant, recessive, and overdominant) were employed, with the additive model assessing the effects of each minor allele on AMD. The best genetic model was selected based on the Akaike information criterion (AIC), favoring models with the lowest AIC values. Bonferroni correction was applied to account for multiple association calculations, resulting in an adjusted significance threshold for multiple comparisons of α = 0.01 (0.05/5, as five different SNPs were analyzed).

## 5. Conclusions

In published databases, we did not uncover any studies examining the serum correlations of TNFRSF1A, TNFRSF1B, and IL1B1 with age-related macular degeneration. In conclusion, identifying genetic variations in *ARMS2*, *VEGFA*, *TNFRSF1A*, *TNFRSF1B*, and *IL1B1* provides valuable insights into the susceptibility to and treatment outcomes for age-related macular degeneration. Our analysis emphasizes the importance of personalized medicine, where genetic profiling could help choose and optimize anti-VEGF therapies. However, despite significant progress, further research through large studies is necessary to confirm these associations and fully comprehend the pharmacogenetic implications. Ultimately, integrating genetic testing into clinical practice could improve the precision and effectiveness of AMD treatment, potentially reducing the burden of this disabling condition. 

## Figures and Tables

**Table 1:** Demographic data of the study.

Characteristic	Early AMD n = 253	Exudative AMD n = 245	Control n = 337	*p*-Value
Gender ^2^ Males, n (%) Females, n (%)	80 (31.6) 173 (68.4)	90 (36.7) 155 (63.3)	115 (34.1) 222 (65.9)	0.522 * 0.515 **
Age years; median (IQR) ^1^	73 (12)	77 (10)	72 (11)	0.117 * <0.001 **

*p*—significance level, significant when *p* < 0.05; IQR—interquartile range. ^1^ Mann–Whitney U test was used to compare age between groups. ^2^ Pearson’s chi-squared test was used to compare the gender distribution between groups. * Early AMD vs. control group; ** Exudative AMD vs. control group.

**Table 2:** Distributions of *VEGFA*, *IL1B*, *TNFRSF1B*, *TNFRSF1A*, and *ARMS2* SNP genotypes and alleles in early and exudative AMD and control groups.

Gene/Marker	Genotype/ Allele	Group			*p*-Value *	*p*-Value **
		Early AMD (n = 253) n (%)	Exudative AMD (n = 245) n (%)	Control (n = 337) n (%)		
*VEGFA* rs3024997	GG GA AA G A	150 (59.3) 86 (34) 17 (6.7) 386 (76.3) 120 (23.7)	155 (63.3) 89 (36.3) 1 (0.4) 399 (81.4) 91 (18.6)	187 (55.5) 129 (38.3) 21 (6.2) 503 (74.6) 171 (25.4)	0.563 0.513	**<0.001** **0.006**
*IL1B* rs1143623	CC CG GG C G	129 (51) 103 (40.7) 21 (8.3) 361 (71.3) 145 (28.7)	134 (54.7) 92 (37.6) 19 (7.8) 360 (73.5) 130 (26.5)	173 (51.3) 143 (42.4) 21 (6.2) 489 (72.6) 185 (27.4)	0.614 0.647	0.445 0.727
*TNFRSF1B* rs1061622	GG GT TT G T	161 (63.6) 79 (31.2) 13 (5.1) 401 (79.2) 105 (20.8)	157 (64.1) 74 (30.2) 14 (5.7) 388 (79.2) 102 (20.8)	220 (65.3) 107 (31.8) 10 (3) 547 (81.2) 127 (18.8)	0.402 0.414	0.225 0.403
*TNFRSF1A* rs4149576	TT TC CC T C	61 (24.1) 123 (48.6) 69 (27.3) 245 (48.4) 261 (51.6)	60 (24.5) 124 (50.6) 61 (24.9) 244 (49.8) 246 (50.2)	89 (26.4) 168 (49.9) 80 (23.7) 346 (51.3) 328 (48.7)	0.589 0.321	0.861 0.604
*ARMS2* rs10490924	GG GT TT G T	116 (45.8) 108 (42.7) 29 (11.5) 340 (67.2) 166 (32.8)	78 (31.8) 106 (43.3) 61 (24.9) 262 (53.5) 228 (46.5)	183 (54.3) 129 (38.3) 25 (7.4) 495 (73.4) 179 (26.6)	0.070 0.019	**<0.001** **<0.001**

*p*—significance level. When Pearson’s chi-squared test was used, Bonferroni-corrected significance level *p* = 0.05/5; *p*-values marked with bold indicate statistically significant *p*-values. * Early AMD vs. control group; ** exudative AMD vs. control group.

**Table 3:** Associations between *VEGFA* (rs3024997), *IL1B* (rs1143623), *TNFRSF1B* (rs1061622), *TNFRSF1A* (rs4149576), *ARMS2* (rs10490924), and exudative AMD.

*VEGFA* (rs3024997)				
Model	Genotype/Allele	OR * (95% CI)	*p*-Value	AIC
Codominant	GA vs. GG AA vs. GG	0.887 (0.617–1.275) 0.047 (0.006–0.366)	0.518 **0.003**	718.602
Dominant	GA + AA vs. GG	0.755 (0.530–1.076)	0.120	733.422
Recessive	AA vs. GG + GA	0.049 (0.006–0.381)	**0.004**	717.021
Overdominant	GA vs. GG + AA	0.987 (0.689–1.413)	0.943	735.844
Additive	A	0.657 (0.480–0.899)	**0.009**	728.805
***IL1B* (rs1143623)**				
**Model**	**Genotype/Allele**	OR * (95% CI)	***p*-value**	**AIC**
Codominant	CG vs. CC GG vs. CC	0.853 (0.593–1.227) 1.195 (0.599–2.384)	0.391 0.613	736.601
Dominant	CG + GG vs. CC	0.898 (0.634–1.270)	0.541	735.476
Recessive	GG vs. CC + CG	1.279 (0.653–2.508)	0.473	735.336
Overdominant	CG vs. CC + GG	0.835 (0.586–1.190)	0.319	734.856
Additive	G	0.973 (0.737–1.284)	0.845	735.811
***TNFRSF1B* (rs1061622)**				
**Model**	**Genotype/Allele**	OR * (95% CI)	***p*-value**	**AIC**
Codominant	GT vs. GG TT vs. GG	0.881 (0.603–1.288) 2.049 (0.847–4.957)	0.514 0.112	734.484
Dominant	GT + TT vs. GG	0.974 (0.678–1.399)	0.888	735.829
Recessive	TT vs. GG + GT	2.133 (0.890–5.116)	0.090	732.910
Overdominant	GT vs. GG + TT	0.844 (0.580–1.229)	0.376	735.063
Additive	T	1.078 (0.795–1.462)	0.627	735.614
***TNFRSF1A* (rs4149576)**				
**Model**	**Genotype/Allele**	OR * (95% CI)	***p*-value**	**AIC**
Codominant	TC vs. TT CC vs. TT	1.140 (0.748–1.737) 1.299 (0.796–2.119)	0.542 0.296	736.752
Dominant	TC + CC vs. TT	1.190 (0.800–1.769)	0.391	735.109
Recessive	CC vs. TT + TC	1.191 (0.796–1.783)	0.394	735.125
Overdominant	TC vs. TT + CC	1.004 (0.711–1.420)	0.980	735.849
Additive	C	1.140 (0.892–1.456)	0.296	734.752
***ARMS2*** (**rs10490924)**				
**Model**	**Genotype/Allele**	OR * (95% CI)	***p*-value**	**AIC**
Codominant	GT vs. GG TT vs. GG	1.768 (1.201–2.603) 5.611 (3.205–9.822)	**0.004** **<0.001**	697.230
Dominant	GT + TT vs. GG	2.377 (1.659–3.404)	**<0.001**	712.955
Recessive	TT vs. GG + GT	4.236 (2.508–7.155)	**<0.001**	703.644
Overdominant	GT vs. GG + TT	1.129 (0.794–1.606)	0.499	735.393
Additive	T	2.200 (1.702–2.846)	**<0.001**	697.423

* OR adjusted for age in exudative AMD group; OR—odds ratio; CI—confidence interval; *p*—significance level, Bonferroni-corrected significance level *p* = 0.05/5; *p*-values marked with bold indicate statistically significant *p*-values; AIC—Akaike information criteria.

**Table 4:** Distributions of *VEGFA*, *IL1B*, *TNFRSF1B*, *TNFRSF1A*, and *ARMS2* SNP genotypes and alleles in early and exudative AMD and control females.

Gene/Marker	Genotype/ Allele	Group			*p*-Value *	*p*-Value **
		Early AMD (n = 173) n (%)	Exudative AMD (n = 155) n (%)	Control (n = 222) n (%)		
*VEGFA* rs3024997	GG GA AA G A	103 (59.5) 60 (34.7) 10 (5.8) 266 (76.9) 80 (23.1)	93 (60) 61 (39.4) 1 (0.6) 247 (79.7) 63 (20.3)	117 (52.7) 92 (41.4) 13 (5.9) 326 (73.4) 118 (26.6)	0.373 0.266	0.022 0.047
*IL1B* rs1143623	CC CG GG C G	93 (53.8) 63 (36.4) 17 (9.8) 249 (72) 97 (28)	84 (54.2) 56 (36.1) 15 (9.7) 224 (72.3) 86 (27.7)	110 (49.5) 99 (44.6) 13 (5.9) 319 (71.8) 125 (28.2)	0.140 0.970	0.152 0.901
*TNFRSF1B* rs1061622	GG GT TT G T	111 (64.2) 55 (31.8) 7 (4) 277 (80.1) 69 (19.9)	97 (62.6) 48 (31) 10 (6.5) 242 (78.1) 68 (21.9)	146 (65.8) 68 (30.6) 8 (3.6) 360 (81.1) 84 (18.9)	0.937 0.718	0.428 0.309
*TNFRSF1A* rs4149576	TT TC CC T C	47 (27.2) 78 (45.1) 48 (27.7) 172 (49.7) 174 (50.3)	33 (21.3) 87 (56.1) 35 (22.6) 153 (49.4) 157 (50.6)	58 (26.1) 116 (52.3) 48 (21.6) 232 (52.3) 212 (47.7)	0.279 0.478	0.555 0.433
*ARMS2* rs10490924	GG GT TT G T	81 (46.8) 75 (43.4) 17 (9.8) 237 (68.5) 109 (31.5)	47 (30.3) 72 (46.5) 36 (23.2) 166 (53.5) 144 (46.5)	120 (54.1) 85 (38.3) 17 (7.7) 325 (73.2) 119 (26.8)	0.342 0.147	**<0.001** **<0.001**

*p*—significance level. When Pearson’s chi-squared test was used, Bonferroni-corrected significance level *p* = 0.05/5; *p*-values marked with bold indicate statistically significant *p*-values. * Early AMD vs. control group; ** exudative AMD vs. control group.

**Table 5:** Associations between *VEGFA* (rs3024997), *IL1B* (rs1143623), *TNFRSF1B* (rs1061622), *TNFRSF1A* (rs4149576), *ARMS2* (rs10490924), and exudative AMD in females.

*VEGFA* (rs3024997)				
Model	Genotype/Allele	OR * (95% CI)	*p*-Value	AIC
Codominant	GA vs. GG AA vs. GG	0.890 (0.561–1.413) 0.074 (0.009–0.624)	0.622 0.017	441.925
Dominant	GA + AA vs. GG	0.777 (0.494–1.221)	0.273	448.552
Recessive	AA vs. GG + GA	0.078 (0.009–0.648)	0.018	440.168
Overdominant	GA vs. GG + AA	0.987 (0.626–1.557)	0.955	449.752
Additive	A	0.680 (0.454–1.018)	0.061	446.194
***IL1B* (rs1143623)**				
**Model**	**Genotype/Allele**	OR * (95% CI)	***p*-value**	**AIC**
Codominant	CG vs. CC GG vs. CC	0.735 (0.458–1.178) 1.567 (0.660–3.718)	0.201 0.308	448.241
Dominant	CG + GG vs. CC	0.831 (0.531–1.299)	0.416	449.094
Recessive	GG vs. CC + CG	1.793 (0.774–4.152)	0.173	447.886
Overdominant	CG vs. CC + GG	0.693 (0.438–1.097)	0.117	447.285
Additive	G	0.988 (0.694–1.408)	0.947	449.751
***TNFRSF1B* (rs1061622)**				
**Model**	**Genotype/Allele**	OR * (95% CI)	***p*-value**	**AIC**
Codominant	GT vs. GG TT vs. GG	0.897 (0.548–1.467) 1.919 (0.670–5.494)	0.664 0.225	449.878
Dominant	GT + TT vs. GG	0.996 (0.625–1.588)	0.986	449.755
Recessive	TT vs. GG + GT	1.986 (0.702–5.618)	0.196	448.067
Overdominant	GT vs. GG + TT	0.857 (0.525–1.393)	0.533	449.364
Additive	T	1.096 (0.747–1.608)	0.638	449.536
***TNFRSF1A* (rs4149576)**				
**Model**	**Genotype/Allele**	OR * (95% CI)	***p*-value**	**AIC**
Codominant	TC vs. TT CC vs. TT	1.472 (0.845–2.564) 1.600 (0.824–3.106)	0.173 0.165	449.329
Dominant	TC + CC vs. TT	1.508 (0.887–2.562)	0.129	447.413
Recessive	CC vs. TT + TC	1.224 (0.716–2.093)	0.461	449.213
Overdominant	TC vs. TT + CC	1.173 (0.749–1.837)	0.484	449.266
Additive	C	1.267 (0.911–1.762)	0.160	447.765
***ARMS2*** (**rs10490924)**				
**Model**	**Genotype/Allele**	OR * (95% CI)	***p*-value**	**AIC**
Codominant	GT vs. GG TT vs. GG	1.979 (1.201–3.261) 5.628 (2.720–11.646)	**0.007** **<0.001**	427.191
Dominant	GT + TT vs. GG	2.550 (1.597–4.071)	**<0.001**	433.851
Recessive	TT vs. GG + GT	3.974 (2.020–7.817)	**<0.001**	432.468
Overdominant	GT vs. GG + TT	1.262 (0.803–1.984)	0.312	448.735
Additive	T	2.265 (1.617–3.172)	**<0.001**	425.703

* OR adjusted for age in exudative AMD group; OR—odds ratio; CI—confidence interval; *p*—significance level, Bonferroni-corrected significance level *p* = 0.05/5; *p*-values marked with bold indicate statistically significant *p*-values; AIC—Akaike information criteria.

**Table 6:** Distributions of *VEGFA*, *IL1B*, *TNFRSF1B*, *TNFRSF1A*, and *ARMS2* SNP genotypes and alleles in early and exudative AMD and control males.

Gene/Marker	Genotype/ Allele	Group			*p*-Value *	*p*-Value **
		Early AMD (n = 80) n (%)	Exudative AMD (n = 90) n (%)	Control (n = 115) n (%)		
*VEGFA* rs3024997	GG GA AA G A	47 (58.8) 26 (32.5) 7 (8.8) 120 (75) 40 (25)	62 (68.9) 28 (31.1) 0 (0) 152 (84.4) 28 (15.6)	70 (60.9) 37 (32.2) 8 (7) 177 (77) 53 (23)	0.890 0.655	0.034 0.058
*IL1B* rs1143623	CC CG GG C G	36 (45) 40 (50) 4 (5) 112 (70) 48 (30)	50 (55.6) 36 (40) 4 (4.4) 136 (75.6) 44 (24.4)	63 (54.8) 44 (38.3) 8 (7) 170 (73.9) 60 (26.1)	0.260 0.395	0.745 0.704
*TNFRSF1B* rs1061622	GG GT TT G T	50 (62.5) 24 (30) 6 (7.5) 124 (77.5) 36 (22.5)	60 (66.7) 26 (28.9) 4 (4.4) 146 (81.1) 34 (18.9)	74 (64.3) 39 (33.9) 2 (1.7) 187 (81.3) 43 (18.7)	0.131 0.357	0.426 0.960
*TNFRSF1A* rs4149576	TT TC CC T C	14 (17.5) 45 (56.3) 21 (26.3) 73 (45.6) 87 (54.4)	27 (30) 37 (41.1) 26 (28.9) 91 (50.6) 89 (49.4)	31 (27) 52 (45.2) 32 (27.8) 114 (49.6) 116 (50.4)	0.220 0.443	0.826 0.842
*ARMS2* rs10490924	GG GT TT G T	35 (43.8) 33 (41.3) 12 (15) 103 (64.4) 57 (35.6)	31 (34.4) 34 (37.8) 25 (27.8) 96 (53.3) 84 (46.7)	63 (54.8) 44 (38.3) 8 (7.0) 170 (73.9) 60 (26.1)	0.121 0.043	**<0.001** <**0.001**

*p*—significance level. When Pearson’s chi-squared test was used, Bonferroni-corrected significance level *p* = 0.05/5; *p*-values marked with bold indicate statistically significant *p*-values. * Early AMD vs. control group; ** exudative AMD vs. control group.

**Table 7:** Associations between *VEGFA* (rs3024997), *IL1B* (rs1143623), *TNFRSF1B* (rs1061622), *TNFRSF1A* (rs4149576), *ARMS2* (rs10490924), and exudative AMD in males.

*VEGFA* (rs3024997)				
Model	Genotype/Allele	OR * (95% CI)	*p*-Value	AIC
Codominant	GA vs. GG AA vs. GG	0.873 (0.479–1.594) -	0.659 **-**	273.752
Dominant	GA + AA vs. GG	0.711 (0.396–1.276)	0.253	280.473
Recessive	AA vs. GG + GA	-	**-**	271.947
Overdominant	GA vs. GG + AA	0.974 (0.536–1.768)	0.930	281.782
Additive	A	0.616 (0.369–1.028)	0.064	278.221
***IL1B* (rs1143623)**				
**Model**	**Genotype/Allele**	OR * (95% CI)	***p*-value**	**AIC**
Codominant	CG vs. CC GG vs. CC	1.049 (0.588–1.870) 0.642 (0.182–2.258)	0.872 0.489	283.201
Dominant	CG + GG vs. CC	0.986 (0.565–1.721)	0.960	281.787
Recessive	GG vs. CC + CG	0.629 (0.183–2.163)	0.462	281.227
Overdominant	CG vs. CC + GG	1.093 (0.619–1.928)	0.759	281.696
Additive	G	0.926 (0.585–1.465)	0.742	281.681
***TNFRSF1B* (rs1061622)**				
**Model**	**Genotype/Allele**	OR * (95% CI)	***p*-value**	**AIC**
Codominant	GT vs. GG TT vs. GG	0.822 (0.450–1.504) 2.526 (0.445–14.345)	0.525 0.296	282.029
Dominant	GT + TT vs. GG	0.904 (0.505–1.619)	0.735	281.675
Recessive	TT vs. GG + GT	2.691 (0.479–15.120)	0.261	280.435
Overdominant	GT vs. GG + TT	0.791 (0.434–1.440)	0.443	281.197
Additive	T	1.017 (0.610–1.695)	0.948	281.786
***TNFRSF1A* (rs4149576)**				
**Model**	**Genotype/Allele**	OR * (95% CI)	***p*-value**	**AIC**
Codominant	TC vs. TT CC vs. TT	0.811 (0.416–1.583) 0.971 (0.465–2.027)	0.540 0.938	283.321
Dominant	TC + CC vs. TT	0.871 (0.472–1.607)	0.658	281.595
Recessive	CC vs. TT + TC	1.101 (0.593–2.043)	0.760	281.697
Overdominant	TC vs. TT + CC	0.823 (0.469–1.444)	0.497	281.327
Additive	C	0.984 (0.680–1.424)	0.933	281.783
***ARMS2*** (**rs10490924)**				
**Model**	**Genotype/Allele**	OR * (95% CI)	***p*-value**	**AIC**
Codominant	GT vs. GG TT vs. GG	1.537 (0.824–2.867) 6.183 (2.495–15.322)	0.177 **<0.001**	265.951
Dominant	GT + TT vs. GG	2.251 (1.270–3.989)	**0.005**	273.879
Recessive	TT vs. GG + GT	5.049 (2.147–11.877)	**<0.001**	265.781
Overdominant	GT vs. GG + TT	0.960 (0.542–1.699)	0.887	281.770
Additive	T	2.205 (1.469–3.310)	**<0.001**	266.193

* OR adjusted for age in exudative AMD group; OR—odds ratio; CI—confidence interval; *p*—significance level, Bonferroni-corrected significance level *p* = 0.05/5; *p*-values marked with bold indicate statistically significant *p*-values; AIC—Akaike information criteria.

**Table 8:** Demographic and clinical parameters.

Characteristic	Non-Responders n = 20	Responders n = 95	*p*-Value
Gender Males, n (%) Females, n (%)	9 (30) 14 (70)	29 (30.5) 66 (689.5)	0.963 *
Age years; mean (SD)	75.4 (7.366)	77.54 (7.784)	0.263 **
**Response parameter**			
VA, median (IQR) Baseline Treated	0.465 (0.45) ^1^ 0.35 (0.35) ^1^	0.28 (0.26) ^2^ 0.375 (0.35) ^2^	0.018 *** 0.408 ***
CRT (μm), median (IQR) Baseline Treated	272.5 (95.25) ^3^ 329 (103) ^3^	320.5 (113) ^4^ 274 (95) ^4^	0.068 *** 0.032 ***

*p*—significance level, significant when *p* < 0.05; *p*-values marked with bold indicate statistically significant *p*-values; IQR—interquartile range; SD—standard deviation; VA—visual acuity; CRT—central macular thickness. * Pearson’s chi-squared test. ** Student’s T test. *** Mann–Whitney U test. ^1^ *p* = 0.028, Wilcoxon signed-rank test; ^2^ *p* < 0.001, Wilcoxon signed-rank test; ^3^ *p* = 0.441, Wilcoxon signed-rank test; ^4^ *p* < 0.001, Wilcoxon signed-rank test.

**Table 9:** Associations between *TNFRSF1B* rs1061622 and response to treatment.

Genetic Model	Genotype/Allele	Non-Responders n = 20 n (%)	Responders n = 95 n (%)	OR (95% CI)	*p*-Value	AIC
Dominant	GT + TT	3 (15)	41 (43.2)	4.302 (1.181; 15.674)	**0.027**	102.065
Additive	T	3 (7.5)	48 (25.3)	3.999 (1.176; 13.602)	**0.026**	101.291

OR—odds ratio; CI—confidence interval; *p*—significance level, significant when *p* < 0.05; *p*-values marked with bold indicate statistically significant *p*-values; AIC—Akaike information criteria.

## Data Availability

The datasets used and/or analyzed during the current study are available from the corresponding author on reasonable request.

## Supplementary Materials

The following supporting information can be downloaded at: https://www.mdpi.com/article/10.3390/ijms25179750/s1.
