# Supplementary material for: The Impact of ARMS2 (rs10490924), VEGFA (rs3024997), TNFRSF1B (rs1061622), TNFRSF1A (rs4149576), and IL1B1 (rs1143623) Polymorphisms and Serum Levels on Age-Related Macular Degeneration Development and Therapeutic Responses
Source: Int J Mol Sci. 2024 Sep 9;25(17):9750. doi: 10.3390/ijms25179750 (PMC11396313; doi:10.3390/ijms25179750)
Supplement: Supplementary file 1 [file ijms-25-09750-s001.zip › ijms-3111354-supplementary.pdf]

Supplementary Materials

Table S1. Associations between *VEGFA* (rs3024997), *IL1B* (rs1143623), *TNFRSF1B* (rs1061622), *TNFRSF1A* (rs4149576), *ARMS2* (rs10490924) and early AMD

***VEGFA* (rs3024997):**				
**Model**	**Genotype/Allele**	**OR (95% CI)**	**p-value**	**AIC**
Codominant	GA *vs.* GG	0.831 (0.587-1.176)	0.296	808.763
	AA *vs.* GG	1.009 (0.514-1.981)	0.979	
Dominant	GA+AA *vs.* GG	0.856 (0.615-1.191)	0.356	807.061
Recessive	AA *vs.* GG+GA	1.084 (0.560-2.100)	0.811	807.857
Overdominant	GA *vs.* GG+AA	0.830 (0.591-1.167)	0.284	806.764
Additive	A	0.916 (0.702-1.195)	0.518	807.494
***IL1B* (rs1143623):**				
**Model**	**Genotype/Allele**	**OR (95% CI)**	**p-value**	**AIC**
Codominant	CG *vs.* CC	0.966 (0.687-1.358)	0.842	808.947
	GG *vs.* CC	1.341 (0.703-2.560)	0.374	
Dominant	CG+GG *vs.* CC	1.014 (0.732-1.405)	0.933	807.907
Recessive	GG *vs.* CC+CG	1.362 (0.727-2.553)	0.335	806.987
Overdominant	CG *vs.* CC+GG	0.932 (0.669-1.297)	0.675	807.737
Additive	G	1.064 (0.819-1.381)	0.641	807.697
***TNFRSF1B* (rs1061622):**				
**Model**	**Genotype/Allele**	**OR (95% CI)**	**p-value**	**AIC**
Codominant	GT *vs.* GG	1.009 (0.708-1.438)	0.961	808.117
	TT *vs.* GG	1.776 (0.760-4.152)	0.185	
Dominant	GT+TT *vs.* GG	1.074 (0.764-1.510)	0.679	807.743
Recessive	TT *vs.* GG+GT	1.771 (0.764-4.107)	0.183	806.119
Overdominant	GT *vs.* GG+TT	0.976 (0.687-1.387)	0.892	807.895
Additive	T	1.128 (0.845-1.505)	0.415	807.251
***TNFRSF1A* (rs4149576):**				
**Model**	**Genotype/Allele**	**OR (95% CI)**	**p-value**	**AIC**
Codominant	TC *vs.* TT	1.068 (0.716-1.594)	0.747	808.857

	CC *vs.* TT	1.258 (0.796-1.990)	0.325	
Dominant	TC+CC *vs.* TT	1.130 (0.775-1.646)	0.526	807.510
Recessive	CC *vs.* TT+TC	1.205 (0.829- 1.750)	0.328	806.961
Overdominant	TC *vs.* TT+CC	0.952 (0.687-1.319)	0.767	807.825
Additive	C	1.122 (0.892-1.411)	0.325	806.943
ARMS2 **(rs10490924):**				
**Model**	**Genotype/Allele**	**OR (95% CI)**	**p-value**	**AIC**
Codominant	GT *vs.* GG<br>TT *vs.* GG	1.321 (0.935-1.866)<br>1.830 (1.021-3.279)	0.115<br>0.042	804.612
Dominant	GT+TT *vs.* GG	1.403 (1.012-1.947)	0.042	803.778
Recessive	TT *vs.* GG+GT	1.616 (0.921-2.834)	0.094	805.104
Overdominant	GT *vs.* GG+TT	1.201 (0.862-1.674)	0.280	806.746
Additive	T	1.340 (1.044-1.722)	0.022	802.627

**- OR adjusted for age in exudative AMD group; OR – odds ratio; CI – confidence interval; p-significance level, Bonferroni corrected significance level p=0.05/5; AIC-Akaike information criteria.*

Table S2. Associations between *VEGFA* (rs3024997), *IL1B* (rs1143623), *TNFRSF1B* (rs1061622), *TNFRSF1A* (rs4149576), *ARMS2* (rs10490924 and early AMD in females

VEGFA **(rs3024997):**				
**Model**	**Genotype/Allele**	**OR (95% CI)**	**p-value**	**AIC**
Codominant	GA *vs.* GG<br>AA *vs.* GG	0.741 (0.487-1.127)<br>0.874 (0.368-2.077)	0.161<br>0.760	543.515
Dominant	GA+AA *vs.* GG	0.757 (0.507-1.132)	0.175	541.647
Recessive	AA *vs.* GG+GA	0.986 (0.422-2.306)	0.975	543.491
Overdominant	GA *vs.* GG+AA	0.750 (0.497-1.132)	0.171	541.608
Additive	A	0.827 (0.593-1.152)	0.261	542.219
IL1B **(rs1143623):**				
**Model**	**Genotype/Allele**	**OR (95% CI)**	**p-value**	**AIC**
Codominant	CG *vs.* CC<br>GG *vs.* CC	0.753 (0.495-1.145)<br>1.547 (0.714-3.351)	0.184<br>0.269	541.559
Dominant	CG+GG *vs.* CC	0.845 (0.567-1.258)	0.407	542.803

Recessive	GG *vs.* CC+CG	1.752 (0.826-3.714)	0.144	541.328
Overdominant	CG *vs.* CC+GG	0.712 (0.473-1.069)	0.102	540.793
Additive	G	0.994 (0.725-1.362)	0.970	543.491
***TNFRSF1B* (rs1061622):**				
**Model**	**Genotype/Allele**	**OR (95% CI)**	**p-value**	**AIC**
Codominant	GT *vs.* GG<br>TT *vs.* GG	1.064 (0.690-1.640)<br>1.151 (0.405-3.269)	0.779<br>0.792	543.362
Dominant	GT+TT *vs.* GG	1.073 (0.708-1.627)	0.740	543.382
Recessive	TT *vs.* GG+GT	1.128 (0.401-3.174)	0.819	543.440
Overdominant	GT *vs.* GG+TT	1.056 (0.688-1.621)	0.805	543.431
Additive	T	1.067 (0.749-1.521)	0.718	543.362
***TNFRSF1A* (rs4149576):**				
**Model**	**Genotype/Allele**	**OR (95% CI)**	**p-value**	**AIC**
Codominant	TC *vs.* TT<br>CC *vs.* TT	0.830 (0.513-1.341)<br>1.234 (0.708-2.150)	0.446<br>0.458	542.940
Dominant	TC+CC *vs.* TT	0.948 (0.605-1.486)	0.816	543.438
Recessive	CC *vs.* TT+TC	1.392 (0.878- 2.208)	0.160	541.520
Overdominant	TC *vs.* TT+CC	0.750 (0.504-1.118)	0.158	541.492
Additive	C	1.105 (0.836-1.461)	0.482	542.998
***ARMS2* (rs10490924):**				
**Model**	**Genotype/Allele**	**OR (95% CI)**	**p-value**	**AIC**
Codominant	GT *vs.* GG<br>TT *vs.* GG	1.307 (0.859-1.988)<br>1.481 (0.715-3.071)	0.211<br>0.291	543.345
Dominant	GT+TT *vs.* GG	1.336 (0.897-1.991)	0.154	541.455
Recessive	TT *vs.* GG+GT	1.314 (0.650-2.656)	0.447	542.915
Overdominant	GT *vs.* GG+TT	1.233 (0.823-1.849)	0.309	542.458
Additive	T	1.253 (0.921-1.705)	0.152	541.431

**- OR adjusted for age in exudative AMD group; OR – odds ratio; CI – confidence interval; p-significance level, Bonferroni corrected significance level p=0.05/5; AIC-Akaike information criteria.*

Table S3. Associations between *VEGFA* (rs3024997), *IL1B* (rs1143623), *TNFRSF1B* (rs1061622), *TNFRSF1A* (rs4149576), *ARMS2* (rs10490924 and early AMD in males

***VEGFA* (rs3024997):**				
**Model**	**Genotype/Allele**	**OR (95% CI)**	**p-value**	**AIC**
Codominant	GA *vs.* GG	1.047 (0.561-1.951)	0.886	267.779
	AA *vs.* GG	1.303 (0.443-3.836)	0.631	
Dominant	GA+AA *vs.* GG	1.092 (0.610-1.954)	0.766	265.923
Recessive	AA *vs.* GG+GA	1.283 (0.446-3.691)	0.645	265.800
Overdominant	GA *vs.* GG+AA	1.015 (0.552-1.868)	0.962	266.009
Additive	A	1.101 (0.704-1.723)	0.672	265.833
***IL1B* (rs1143623):**				
**Model**	**Genotype/Allele**	**OR (95% CI)**	**p-value**	**AIC**
Codominant	CG *vs.* CC	1.591 (0.880-2.877)	0.125	265.320
	GG *vs.* CC	0.875 (0.246-3.110)	0.836	
Dominant	CG+GG *vs.* CC	1.481 (0.835-2.627)	0.180	264.202
Recessive	GG *vs.* CC+CG	0.704 (0.205-2.422)	0.578	265.691
Overdominant	CG *vs.* CC+GG	1.614 (0.906-2.875)	0.104	263.363
Additive	G	1.234 (0.773-1.971)	0.378	265.233
***TNFRSF1B* (rs1061622):**				
**Model**	**Genotype/Allele**	**OR (95% CI)**	**p-value**	**AIC**
Codominant	GT *vs.* GG	0.911 (0.489-1.697)	0.768	263.954
	TT *vs.* GG	4.440 (0.861-22.890)	0.075	
Dominant	GT+TT *vs.* GG	1.083 (0.599-1.958)	0.792	265.942
Recessive	TT *vs.* GG+GT	4.581 (0.900-23.309)	0.067	262.041
Overdominant	GT *vs.* GG+TT	0.835 (0.452-1.544)	0.566	265.679
Additive	T	1.263 (0.767-2.081)	0.359	265.171
***TNFRSF1A* (rs4149576):**				
**Model**	**Genotype/Allele**	**OR (95% CI)**	**p-value**	**AIC**
Codominant	TC *vs.* TT	1.916 (0.908-4.043)	0.088	264.938
	CC *vs.* TT	1.453 (0.629-3.357)	0.382	

Dominant	TC+CC *vs.* TT	1.740 (0.856-3.534)	0.126	263.578
Recessive	CC *vs.* TT+TC	0.923 (0.485- 1.757)	0.808	265.952
Overdominant	TC *vs.* TT+CC	1.558 (0.877-2.767)	0.130	263.709
Additive	C	1.171 (0.781-1.755)	0.445	265.425
ARMS2 **(rs10490924):**				
**Model**	**Genotype/Allele**	**OR (95% CI)**	**p-value**	**AIC**
Codominant	GT *vs.* GG	1.350 (0.732-2.489)	0.336	263.833
	TT *vs.* GG	2.700 (1.008-7.233)	0.048	
Dominant	GT+TT *vs.* GG	1.558 (0.877-2.767)	0.130	263.709
Recessive	TT *vs.* GG+GT	2.360 (0.918-6.072)	0.075	262.757
Overdominant	GT *vs.* GG+TT	1.133 (0.633-2.029)	0.675	265.835
Additive	T	1.534 (0.997-2.361)	0.051	262.167

**- OR adjusted for age in exudative AMD group; OR – odds ratio; CI – confidence interval; p-significance level, Bonferroni corrected significance level p=0.05/5; AIC-Akaike information criteria.*

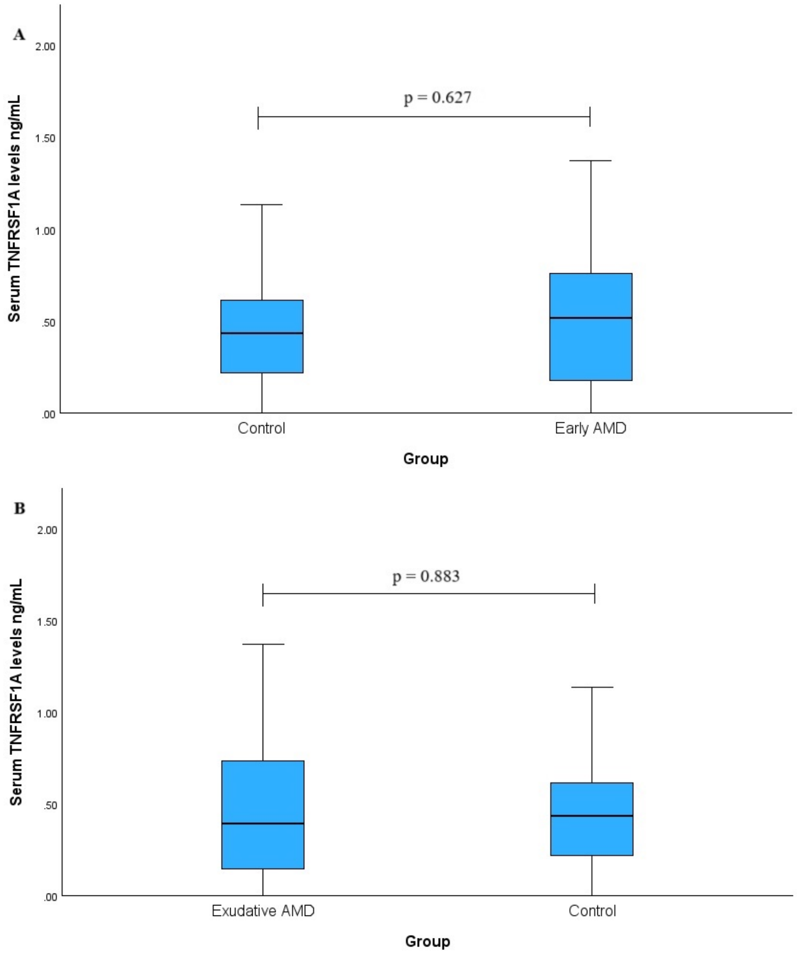

Figure S1. Serum TNFRSF1A levels were measured in patients with early AMD vs. control group (A) and exudative AMD vs. control groups (B). *Mann-Whitney U test was used.*

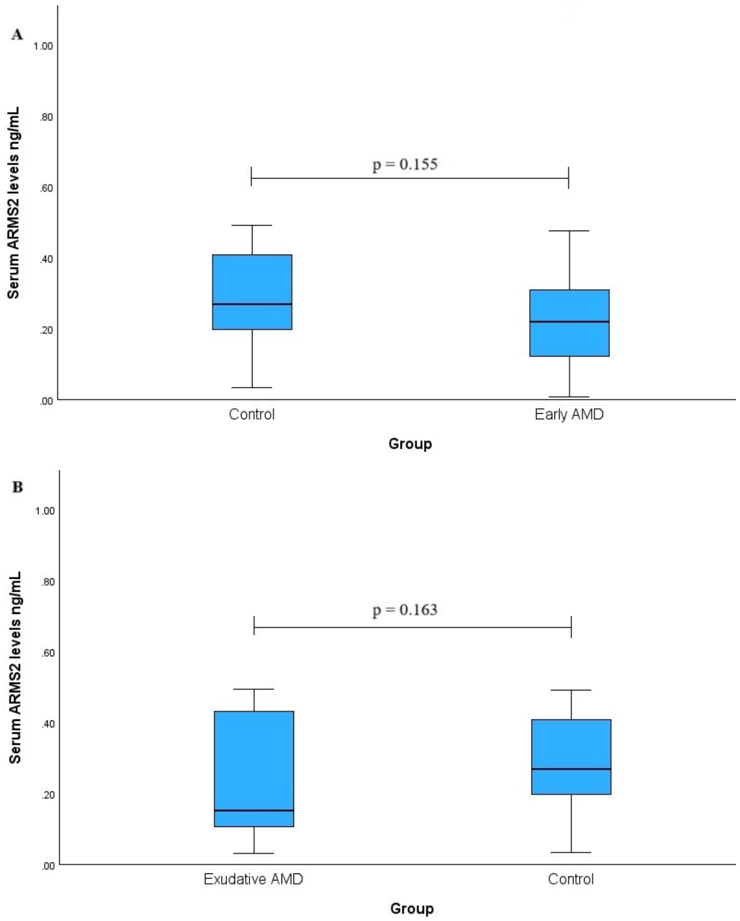

Figure S2. Serum ARMS2 levels were measured in patients with early AMD *vs.* control group (A) and exudative AMD *vs.* control groups (B). *Mann-Whitney U test was used.*

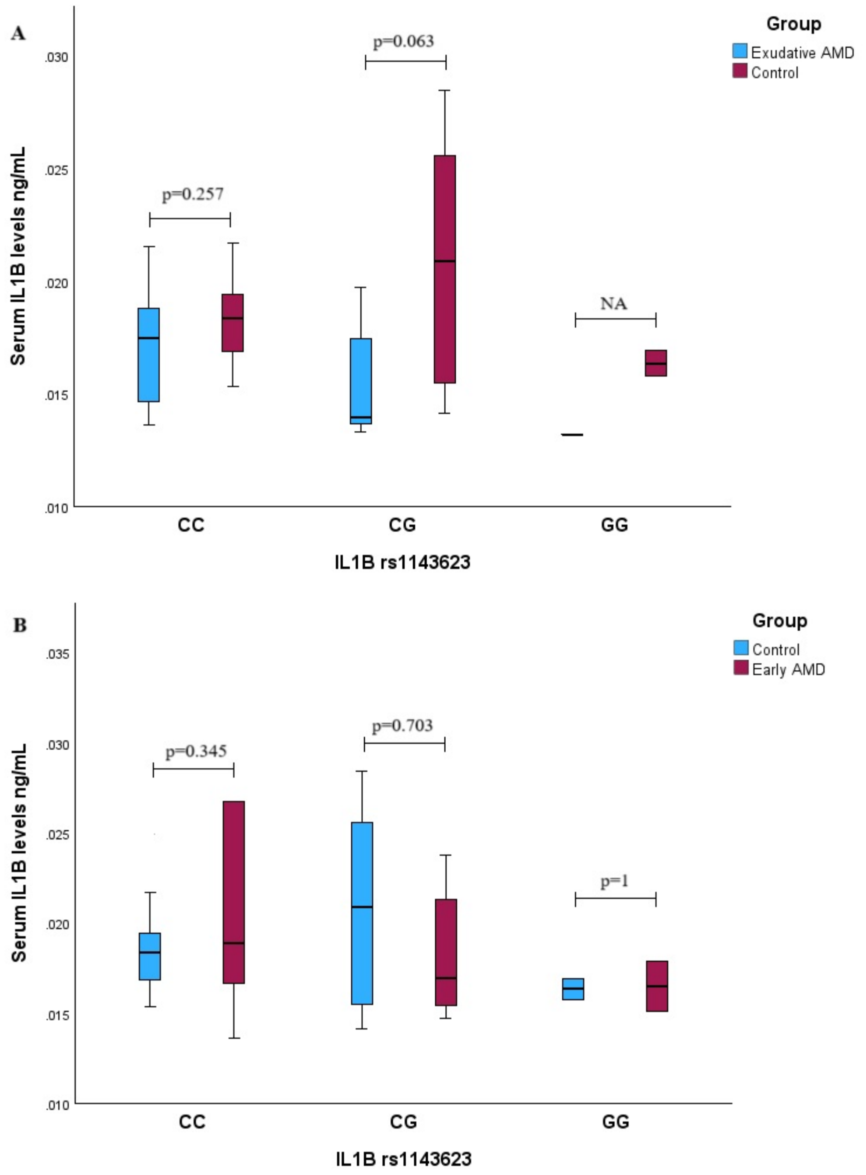

Figure S3. Serum IL1B levels were measured in patients with exudative AMD *vs.* control group and compared between *IL1B* rs1143623 genotypes (A) and between early AMD *vs.* control group (B). *Mann-Whitney U test was used.*

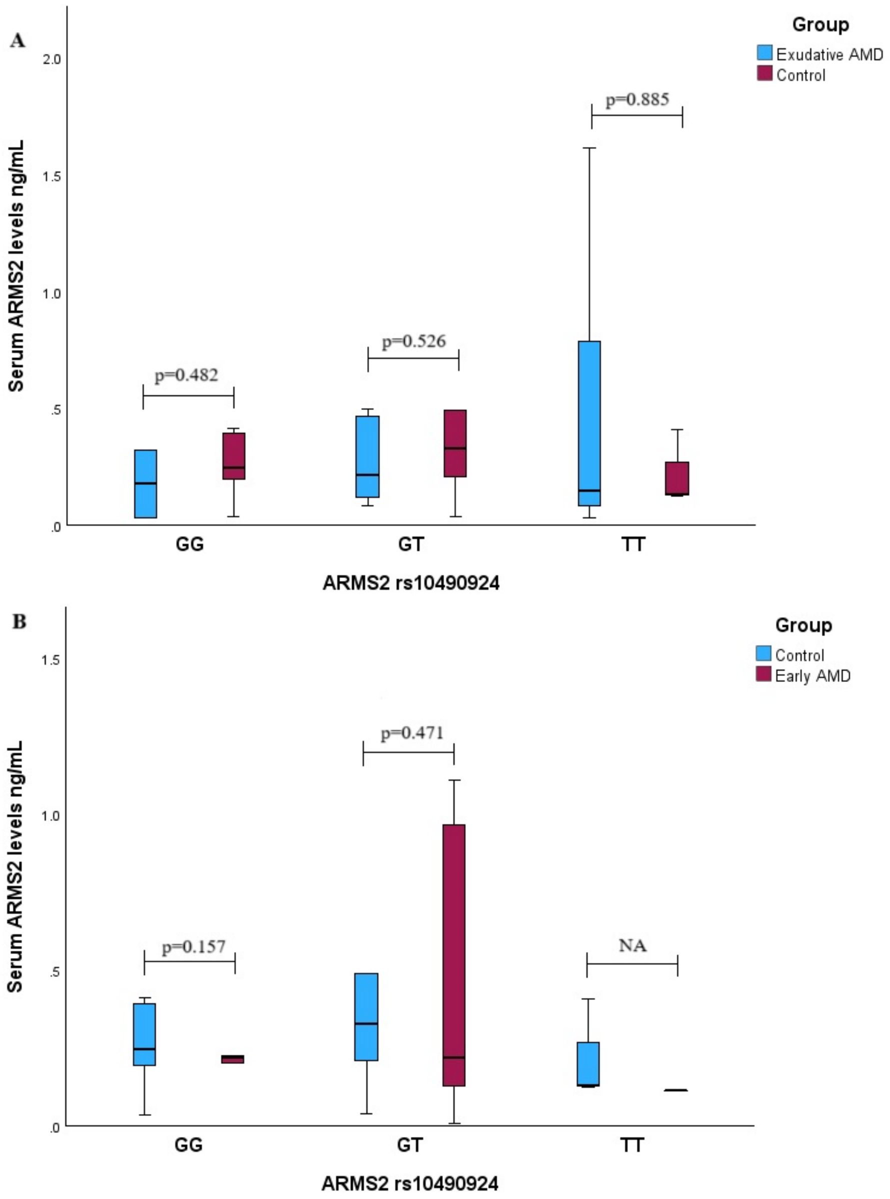

Figure S4. Serum ARMS2 levels were measured in patients with exudative AMD *vs.* control group and compared between *ARMS2* rs10490924 genotypes (A) and between early AMD *vs.* control group (B). *Mann-Whitney U test was used.*
